# Retraction: *Manilkara zapota* L. extract topical ointment application to skin wounds in rats speeds up the healing process

**DOI:** 10.3389/fphar.2026.2021932

**Published:** 2026-10-15

**Authors:** Frontiers Editorial Office

The journal retracts the 29 June 2023 article cited above.

Following publication, concerns were raised regarding the scientific validity of the article and the integrity of the images in the published figures. An investigation was conducted in accordance with Frontiers’ policies, which concluded that the complaints were valid and that the article does not meet the standards of editorial and scientific soundness for Frontiers in Pharmacology; therefore, the article has been retracted.

This retraction was approved by the Chief Editors of Frontiers in Pharmacology and the Chief Executive Editor of Frontiers. The authors do not agree with this retraction.

Frontiers would like to thank Dr. Elisabeth Bik for contacting the journal regarding the published article.

